# THE BLUES AND OLDER MINORITY MUSICIANS: MORE THAN JUST MUSIC XXXI

**DOI:** 10.1093/geroni/igae098.2241

**Published:** 2024-12-31

**Authors:** John Migliaccio, Michael Marcus

**Affiliations:** Chair; Co-Chair

## Abstract

The Blues may have emerged in the Mississippi Delta but its influence and music is mightily represented today in the Puget Sound! Seattle’s Blues Bash, Blues Awards, and the Rhapsody Project make intergenerational cultural connections between the Blues and the community by tuning into cultural heritage exploration and social justice. With a lively array of bars, clubs, and iconic Blues musicians with insights into their lives, influences, resilience and productive aging this popular session draws an enthusiastic crowd of GSA members for a lecture/interview and “mini-performance” with leading older musician. But Wait! Theres more- later that evening, a full performance and party at a local Blues joint guarantees good music, dancing, libations, and Blues swag. This is a “don’t miss” combo with 31 years of GSA history.

